# Comparison between various indices of exposure to traffic-related air pollution and their impact on respiratory health in adults

**DOI:** 10.1136/oem.2007.037846

**Published:** 2008-01-18

**Authors:** G Cesaroni, C Badaloni, D Porta, F Forastiere, C A Perucci

**Affiliations:** Department of Epidemiology, Rome E Health Authority, Rome, Italy

## Abstract

**Objective:**

To evaluate the association of different indices of traffic-related air pollution (self-report of traffic intensity, distance from busy roads from geographical information system (GIS), area-based emissions of particulate matter (PM), and estimated concentrations of nitrogen dioxide (NO_2_) from a land-use regression model) with respiratory health in adults.

**Methods:**

A sample of 9488 25–59-year-old Rome residents completed a self-administered questionnaire on respiratory health and various risk factors, including education, occupation, housing conditions, smoking, and traffic intensity in their area of residence. The study used GIS to calculate the distance between their home address and the closest high-traffic road. For each subject, PM emissions in the area of residence as well as estimated NO_2_ concentrations as assessed by a land-use regression model (R^2^ value = 0.69), were available. Generalised estimating equations (GEE) were used to analyse the association between air pollution measures and prevalence of “ever” chronic bronchitis, asthma, and rhinitis taking into account the effects of age, gender, education, smoking habits, socioeconomic position, and the correlation of variables for members of the same family.

**Results:**

Three hundred and ninety seven subjects (4% of the study population) reported chronic bronchitis, 472 (5%) asthma, and 1227 (13%) rhinitis. Fifteen per cent of subjects reported living in high traffic areas, 11% lived within 50 m of a high traffic road, and 28% in areas with estimated NO_2_ greater than 50 μg/m^3^. Prevalence of asthma was associated only with self-reported traffic intensity whereas no association was found for the other more objective indices. Rhinitis, on the other hand, was strongly associated with all traffic-related indicators (eg, OR = 1.13, 95% CI: 1.04 to 1.22 for 10 μg/m^3^ NO_2_), especially among non-smokers.

**Conclusions:**

Indices of exposure to traffic-related air pollution are consistently associated with an increased risk of rhinitis in adults, especially among non-smokers. The results for asthma are weak, possibly due to ascertainment problems.

During the last two decades a substantial body of epidemiological research has shown that outdoor air pollution, and in particular traffic-related air pollution, is a contributing cause of premature mortality and morbidity.^1^ ^2^ Several studies have reported adverse respiratory effects from traffic exposures among children,^3^^–^5 but the evidence of an effect among adults in the general population is more limited.^6^^–^9 An increased risk of persistent wheeze was associated with living within 50 m of a major roadway in a US veterans study,^8^ while prevalence of chronic bronchitis, respiratory symptoms and hay fever was increased among adults living at busy roads in Germany.^9^ Swiss investigators have used data from the Swiss Cohort Study on Air Pollution and Lung Diseases in Adults (SAPALDIA), conducted in 1991 and 2002, to study the association between traffic exposures and prevalence of respiratory symptoms in a 12-month period in a random adult population sample. They found that non-smokers living within 20 m of a main street had an increased risk of regular phlegm and wheezing.^7^ A positive association with a sensitisation to pollen was also seen in the same study.^10^ In a very recent investigation in the USA, distance from busy roads has been associated with reduced lung function among adults.^11^

Exposure assessment in studies addressing long-term effects of air pollution is a critical issue since urban fixed air pollution monitors do not differentiate the geographical variability of the exposure. Some studies in the past, especially among children, relied on subjective measures of traffic air pollution.^3^ ^12^ However, misclassification of exposure is a common phenomenon and reporting bias could be significant, especially when exposure and outcome are collected from the same individuals. Jacquemin and colleagues have recently indicated that female gender, respiratory symptoms and rhinitis, high education, non-smoking and exposure to environmental tobacco smoke are associated with higher reports of annoyance from air pollution.^13^ Kuehni and colleagues suggested that reporting biases could explain the association between self-reported traffic exposure and respiratory symptoms in children.^14^ In recent years, the development of geographical information system (GIS) techniques, intense ambient monitoring of air pollutants, air dispersion modelling and land- use regression modelling has improved the available tools for better exposure assessment and more reliable indicators.^5^ ^7^ ^15^ ^17^ Still more research is needed to understand the role of traffic air pollution on adult respiratory health, and to clarify the specific exposure indicator most sensitive in detecting a health effect.

The objective of this study was to evaluate the association between long-term exposure to air pollution, estimated by different indices of traffic air pollution (self-reported traffic intensity, GIS-derived proximity measurements to busy roads, emissions data, estimates from a land-use regression model), and prevalence of chronic bronchitis, asthma, and rhinitis in adults.

## MATERIALS AND METHODS

### Study population

Data were derived from the Italian Studies on Respiratory Disorders in Childhood and Environment (SIDRIA) study, an extension of the International Study on Asthma and Allergies in Childhood (ISAAC) initiative in Italy. A cross-sectional survey was carried out between October 1994 and March 1995 in eight centres of northern and central Italy using standardised questionnaires (response rate = 94%). Details of the survey have been extensively reported.^18^ Parents of 7013 subjects (first and second graders from a representative sample of primary schools, and adolescents in the third year of a representative sample of junior high schools) answered a self-administered questionnaire on the child’s health status, as well as their personal respiratory health status and various risk factors, including education, occupation, housing conditions, smoking habits, and traffic intensity in their area of residence. An area-based index of socioeconomic position was assigned to each family. The index was developed using the 2001 Census data on education, occupation, housing tenure, family composition, and immigration by census block (500 average number of residents).^19^ A record linkage was performed with the Rome Municipal Registry Office Database to collect the residential history of parents who lived in Rome with their children at the time of the survey. We were able to identify 5104 fathers (76%) and 5668 mothers (83%). For this study we selected 9488 subjects aged 25–59 years who had been residents in the same place for at least 3 years before the interview.

### Exposure indices

*Self-reported traffic intensity* in the area of residence was self-reported (traffic absent, low, moderate, high).

*GIS indices* were developed for each individual. We geocoded each subject’s residence at the time of the survey. To locate the address on the map we used the Environmental Systems Research Institute, Inc. (ESRI) Italian road network. Eighty subjects (0.8% of the study population) had missing address data. The Municipal Office of Rome gave us the data for all high traffic roads (HTRs) in Rome. We defined two different GIS indicators: the distance from the residence to the nearest HTR, and the total length of HTR segments within a 200 m buffer zone. Similarly to the SAPALDIA study,^7^ we applied buffers of different radii (50, 100 and 200 m) to the residences and intersected the buffers with the list of HTRs to create a categorical variable indicating the distance to HTRs (HTR more than 200 m, between 100 and 200 m, between 50 and 100 m, less than 50 m away). We calculated a four-category variable in metres from home to HTR as the tertiles of the sum of segments’ lengths within the 200 m buffer (none, low <416 m, medium 416–798 m, high >798 m of HTR within 200 m from home).

We collected and stored all geographical variables using ArcGis 9.1 (ESRI, Redlands, California, USA). We used the Word Geodetic System of 1984 with the Universal Transverse Mercator 33N as the coordinate system and map projection.

*Emissions (kg/km^2^) of particulate matter* from traffic were estimated by the Mobility Agency of Rome (STA) for 164 geographical areas of the city. The emissions were estimated using the Transport Energy and Environment (TEE) model developed by the National Research Centre for Energy and Environment (ENEA). The estimate is based on Computer Programme to Calculate Emissions from Road Transport (COPERT II) methodology. This methodology takes many parameters into account, which include vehicle park (number of vehicles per vehicle category, age distribution of the vehicle park per vehicle category), driving conditions (hot and cold annual mileage per vehicle class, annual mileage per road class, average speed of vehicles), emission factors (per vehicle class, per production year, per road class), fuel consumption (per fuel type, per vehicle category), fuel properties, road gradients, and climatic conditions.^20^ Road network graphs from 2001 and average weekday transit during 7:45–8:45 — morning peak traffic hour in 2002 — were used to calculate daily vehicular emissions. Emissions data from earlier time periods were not available. For estimating particulate matter (PM) exhaust emissions, diesel passenger cars, diesel light and heavy duty vehicles were used. Ordinary kriging was applied to provide a smooth surface of emissions, in order to have a continuous geographic description of emissions data. We attributed the average PM exhaust emissions at each subject’s census block of residence, and a categorical variable defined as the quartiles of PM emissions.

A *land-use regression model* to estimate nitrogen dioxide (NO_2_) concentrations was developed for the city, which has been described elsewhere.^15^ Briefly, traffic-related air pollution data were available in the form of NO_2_ measurements performed at the locations of the 70 schools selected for the study. Three Palmes tubes per school measured outdoor pollution simultaneously over three 7-day periods in June 1995, November 1995 and March 1996. The values for the three passive dosimeters in each period were averaged and then the school mean NO_2_ concentration over the entire period was computed as an estimate of the annual mean level. Only sites with complete data coverage throughout the measurement period and which were situated in the region were included, leaving 68 sites for the analysis. Using a multiple linear regression, distance from busy roads, size of census block, number of residents in the census block and population density fitted the NO_2_ data with a determination coefficient (R^2^) of 0.686. For each subject we estimated NO_2_ exposure at their census block of residence using the land-use regression model. We attributed the continuous variable as well as the categorical variable given by the quartiles of its distribution.

Finally, we created a score of traffic air pollution exposure as the sum of the values for four categorical objective measures of traffic air pollution: the distance to HTR, the metres of HTR from home, the quartiles of PM emissions, and the quartiles of NO_2_ concentrations at census block of residence (each variable had a value of 1 for low exposure and 4 for high exposure). The score index ranged from 4 (minimum level of traffic exposure) to 16 (for those living within 50 m of an HTR, with more than 798 m of HTR within 200 m of home, with the highest quartile of PM emissions, and the highest quartile of estimated NO_2_), and we created a categorical variable using the quartile of the distribution: very low (4–5), low (6–7), intermediate (8–10) and high (11–16).

### Outcome measures

Adults under study reported whether they had ever had one or more of the following conditions: chronic bronchitis or emphysema, asthma and rhinitis. More detailed information on the date of onset was not available.

### Statistical analysis

Spearman’s rank correlation coefficients and Pearson’s correlation coefficients were used to measure the correlation between categorical and continuous indices of exposure to traffic air pollution at each family address. We used regression analysis to evaluate the association between each traffic-related indicator and all other covariates, and to calculate p values for trend while accounting for the dependency of members of the same family.

We used Generalised Estimating Equations (GEE) with a logit link to assess the association between traffic exposures and respiratory diseases, adjusting for sex, age (as a continuous variable), smoking habits, and educational level, and taking account of the correlation of data for members of the same family. We used the Wald test to calculate the p for trend for categorical variables in the regression models.

## RESULTS

Table 1 shows the characteristics of the study population according to self-reported traffic, distance from HTRs, metres of HTRs within 200 m of home, area-based PM emissions, and estimated NO_2_. Mean participants’ age was 40 years, 45% had less than 9 years of education, 12% had managerial jobs, 31% were never smokers and 40% were current smokers. Four per cent of the study population (397 subjects) reported to have suffered chronic bronchitis or emphysema, 5% (472) asthma, and 13% (1227) rhinitis. A total of 15% of the participants reported living in high traffic areas. The study subjects lived, at the time of the survey, at an average distance of 463 m from an HTR, with an average of 684 m of HTRs within 200 m of their residence, and in areas with 0.12 kg/km^2^ emissions of PM. A total of 11% of the subjects lived within 50 m of an HTR. Twenty-eight and 40% of the study population lived in areas with estimated NO_2_ greater than 50 and 48.3 μg/m^3^. All indices of exposure to air pollution were directly correlated with high socioeconomic position (measured as educational and occupational level, and area-based index). In addition, a crude association with some air pollution indices was found for age (higher exposure at older age), humidity and rhinitis.

**Table 1:** Characteristics of the study population according to self-reported traffic, distance from high traffic roads (HTRs), metres of HTRs within 200 m from home, particulate matter (PM) emissions, and estimated nitrogen dioxide (NO_2_) from the land-use regression model

	n	%	Self-reported intense traffic (%)	Distance from HTR, mean (SD)	Metres of HTR within 200 m from home, mean (SD)	PM emissions (kg/m^2^), mean (SD)	Estimated NO_2 _(μg/m^3^), mean (SD)
Gender							
Males	4491	47.3	15.0	464 (531)	684 (474)	0.120 (0.081)	45.3 (8)
Females	4997	52.7	15.0	463 (536)	684 (466)	0.120 (0.081)	45.5 (8)
p Value			0.891	0.870	0.958	0.448	0.043
Age (years)							
25–34	1722	18.2	12.2	598 (610)	677 (411)	0.098 (0.080)	43.2 (8)
35–44	5539	58.4	15.2	452 (535)	668 (470)	0.121 (0.080)	45.4 (8)
45+	2228	23.5	16.4	389 (438)	723 (491)	0.136 (0.082)	47.0 (8)
p			0.003	<0.001	0.037	<0.001	<0.001
Education (years)							
<9	4282	45.1	11.6	559 (623)	688 (462)	0.106 (0.079)	43.6 (8)
9–13	3690	38.9	17.2	414 (453)	699 (477)	0.126 (0.082)	46.2 (8)
>13	1356	14.3	19.5	292 (328)	641 (463)	0.148 (0.076)	49.1 (7)
p trend			<0.001	<0.001	0.153	<0.001	<0.001
Occupation							
Managerial/professional	1112	11.7	18.2	349 (395)	628 (454)	0.132 (0.079)	47.3 (8)
Other non-manual	3785	39.9	16.9	418 (484)	700 (482)	0.130 (0.082)	46.4 (8)
Manual labour	1248	13.2	13.3	543 (594)	690 (466)	0.109 (0.079)	44.1 (8)
Other or unemployed	1147	12.1	12.2	498 (547)	650 (434)	0.111 (0.080)	44.3 (8)
Housewife	2091	22.0	12.2	534 (605)	702 (471)	0.108 (0.081)	43.9 (8)
p trend*			<0.001	<0.001	0.750	<0.001	<0.001
Area-based SEP							
High	1698	17.9	19.6	272 (261)	649 (495)	0.157 (0.076)	50.3 (6)
Medium high	1688	17.8	21.0	310 (361)	748 (512)	0.147 (0.081)	48.2 (8)
Intermediate	1657	17.5	19.9	398 (483)	689 (459)	0.136 (0.082)	46.4 (8)
Medium low	1824	19.2	11.4	511 (549)	683 (414)	0.098 (0.076)	42.4 (8)
Low	2432	25.6	7.1	709 (669)	589 (388)	0.083 (0.066)	41.6 (7)
p trend			<0.001	<0.001	0.144	<0.001	<0.001
Smoking habit							
Non-smoker	2977	31.4	14.8	440 (504)	688 (474)	0.121 (0.083)	45.4 (8)
Ex-smoker	2641	27.8	15.8	462 (548)	683 (465)	0.120 (0.080)	45.7 (8)
Current smoker	3822	40.3	14.2	481 (545)	682 (469)	0.119 (0.081)	45.2 (8)
p trend			0.478	0.006	0.784	0.251	0.194
Humidity or moulds							
No	9010	95.0	15.0	460 (530)	684 (468)	0.121 (0.081)	45.5 (8)
Yes	411	4.3	13.1	551 (581)	698 (511)	0.100 (0.079)	43.0 (8)
p Value			0.451	0.023	0.820	<0.001	0.936
Chronic bronchitis or emphysema							
No	9091	95.8	14.9	463 (535)	683 (470)	0.120 (0.081)	45.4 (8)
Yes	397	4.2	17.5	466 (492)	712 (468)	0.119 (0.077)	45.3 (8)
p Value			0.183	0.919	0.491	0.858	0.818
Asthma							
No	9016	95.0	14.8	465 (537)	685 (469)	0.120 (0.081)	45.4 (8)
Yes	472	5.0	19.1	442 (463)	659 (472)	0.121 (0.081)	45.7 (8)
p Value			0.016	0.347	0.490	0.866	0.379
Rhinitis							
No	8261	87.1	14.7	468 (539)	681 (465)	0.119 (0.081)	45.3 (8)
Yes	1227	12.9	17.3	431 (495)	701 (499)	0.127 (0.081)	46.4 (8)
p Value			0.018	0.015	0.434	0.001	<0.001

SEP, socioeconomic position.Totals may vary because of missing information.

p for testing homogeneity.

*Housewives are excluded.

Table 2 shows the correlation among the different traffic exposure indices. The highest correlation of air-pollution continuous variables was between estimated NO_2_ and PM emissions (0.86), followed by estimated NO_2_ and distance to HTR (−0.48). The categorical traffic variable had a correlation with the other categorical variables that ranged between 0.32 and 0.49. There was a statistically significant association between self-reported traffic and all continuous measures of air pollution considered (p<0.001): those who reported high traffic at their home address had a mean distance from an HTR of 188 m versus 756 m for those who reported the absence of traffic; they had more mean metres of HTR within 200 m of home (854 m vs 602 m of those who reported living on roads with no traffic); three times more PM emissions (0.186 vs 0.067 kg/m^2^); and a higher mean estimated NO_2_ (51 μg/m^3^ vs 39 μg/m^3^).

**Table 2:** Cross-comparison of indices of exposure among the study population: self-reported traffic, distance from high traffic roads (HTRs), metres of HTR 5 within 200 m from home, particulate matter (PM) emissions, estimated nitrogen dioxide (NO_2_)

	n	%	Self-reported intense traffic (%)	Distance from HTR, mean (SD)	Metres of HTR within 200 m from home, mean (SD)	PM emissions (kg/m^2^), mean (SD)	Estimated NO_2_ (μg/m^3^), mean (SD)
Self-reported traffic							
Absent	1846	19.5	–	756 (689)	602 (408)	0.067 (0.062)	39.2 (8)
Low	3094	32.6	–	521 (491)	575 (401)	0.101 (0.073)	43.9 (7)
Intermediate	3063	32.3	–	349 (388)	659 (444)	0.140 (0.075)	47.8 (7)
High	1415	14.9	–	188 (387)	854 (533)	0.186 (0.074)	51.5 (6)
p Value				<0.001	<0.001	<0.001	<0.001
Distance from HTRs							
>200 m	5898	62.2	7.4	684 (566)	–	0.099 (0.074)	43.4 (8)
100–200 m	1684	17.7	18.5	149 (29)	431 (329)	0.149 (0.080)	48.2 (7)
50–100 m	813	8.6	25.1	76 (15)	829 (404)	0.154 (0.080)	49.3 (7)
<50 m	1014	10.7	44.4	11 (17)	988 (486)	0.167 (0.080)	49.5 (6)
p Value			<0.001	–	–	<0.001	<0.001
Metres of HTR within 200 m from home							
None	5898	62.2	7.4	684 (566)	–	0.099 (0.074)	43.4 (8)
Low (<416 m)	1169	12.3	18.1	140 (54)	232 (127)	0.142 (0.077)	48.2 (7)
Medium (416–798 m)	1177	12.4	25.6	89 (52)	613 (111)	0.148 (0.082)	49.3 (7)
High (>798 m)	1165	12.3	38.9	47 (46)	1209 (386)	0.176 (0.077)	49.5 (6)
p Value			<0.001	–	–	<0.001	<0.001
PM emissions (quartiles)							
1st	2406	25.4	2.7	825 (746)	595 (309)	0.023 (0.014)	34.6 (4)
2nd	2357	24.8	8.6	457 (456)	543 (377)	0.087 (0.018)	45.4 (4)
3rd	2312	24.4	16.0	328 (302)	638 (495)	0.143 (0.022)	47.9 (3)
4th	2311	24.4	33.2	225 (230)	829 (495)	0.232 (0.043)	54.1 (4)
p Value			<0.001	<0.001	<0.001	–	<0.001
Estimated NO_2 _(quartiles, μg/m^3^)							
1st (21.0–37.3)	2346	24.7	2.7	840 (770)	595 (308)	0.028 (0.028)	33.7 (3)
2nd (37.3–47.3)	2291	24.1	6.2	552 (444)	574 (405)	0.098 (0.042)	44.6 (3)
3rd (47.3–50.3)	2354	24.8	16.2	261 (211)	594 (435)	0.127 (0.032)	48.6 (1)
4th (50.3–62.6)	2366	24.9	35.0	194 (157)	825 (517)	0.227 (0.047)	54.6 (4)
p Value			<0.001	<0.001	<0.001	<0.001	–

Totals may vary because of missing information.

Table 3 reports the association of individual factors with the three health outcome variables. Chronic bronchitis or emphysema was strongly associated with smoking habits (current smokers had OR = 2.68, 95% CI: 2.00 to 3.60 compared to never smokers), older age (p<0.001), low education (OR = 1.78, 95% CI: 1.25 to 2.52 for those with the lowest level of education), low area-based socioeconomic index, and the presence of humidity or moulds at home (OR = 1.98, 95% CI: 1.34 to 2.91). Subjects who suffered from asthma were more likely to be highly educated, and have humidity or moulds in their house (OR = 1.55, 95% CI: 1.04 to 2.31). A high prevalence of rhinitis was associated with higher levels of education.

**Table 3:** Association between personal characteristics and respiratory diseases

	Chronic bronchitis or emphysema (n = 397)		Asthma (n = 472)		Rhinitis (n = 1227)	
	%	OR 95% CI	%	OR 95% CI	%	OR 95% CI
Smoking habit						
Non-smoker	2.1	1.00	5.3	1.00	16.1	1.00
Ex-smoker	4.5	2.10 (1.53 to 2.89)	5.5	1.08 (0.85 to 1.37)	13.8	0.83 (0.71 to 0.97)
Current smoker	5.5	2.64 (1.96 to 3.56)	4.3	0.87 (0.69 to 1.09)	9.9	0.59 (0.51 to 0.68)
p Value		<0.001		0.199		<0.001
Education (years)						
<9	4.8	1.75 (1.22 to 2.50)	4.0	0.59 (0.44 to 0.78)	10.0	0.50 (0.42 to 0.59)
9–13	3.8	1.43 (0.99 to 2.06)	5.4	0.78 (0.60 to 1.02)	14.2	0.71 (0.60 to 0.85)
>13	2.9	1.00	6.6	1.00	18.1	1.00
p Value		0.001		<0.001		<0.001
Occupation						
Managerial/professional	4.2	1.00	5.3	1.00	16.1	1.00
Other non-manual	4.0	0.87 (0.61 to 1.26)	5.2	1.02 (0.73 to 1.43)	14.3	0.94 (0.76 to 1.15)
Manual labour	5.1	1.00 (0.65 to 1.54)	4.5	1.08 (0.70 to 1.67)	10.2	0.78 (0.59 to 1.03)
Other or unemployed	6.4	1.39 (0.91 to 2.12)	4.9	1.06 (0.70 to 1.61)	11.4	0.79 (0.60 to 1.03)
Housewife	2.8	0.67 (0.41 to 1.10)	4.7	0.98 (0.63 to 1.53)	11.5	0.76 (0.58 to 1.01)
Area-based SEP						
High	3.5	1.00	5.3	1.00	14.6	1.00
Medium high	4.2	1.09 (0.75 to 1.59)	5.6	1.11 (0.81 to 1.51)	15.7	1.20 (0.99 to 1.46)
Intermediate	3.3	0.87 (0.58 to 1.31)	5.0	1.05 (0.76 to 1.46)	13.0	1.02 (0.82 to 1.25)
Medium low	4.6	1.29 (0.89 to 1.87)	3.8	0.82 (0.58 to 1.15)	12.1	0.98 (0.79 to 1.20)
Low	5.2	1.44 (1.00 to 2.08)	5.2	1.17 (0.85 to 1.61)	10.9	0.92 (0.74 to 1.14)
p trend		0.027		0.787		0.121
Humidity or moulds at home						
No	4.0	1.00	4.8	1.00	13.0	1.00
Yes	7.5	1.99 (1.34 to 2.97)	7.1	1.55 (1.04 to 2.31)	10.5	0.80 (0.57 to 1.11)

OR, odds ratio adjusted for age, sex, smoking habit and educational level.

Table 4 shows the association of indices of traffic-related pollution with respiratory diseases adjusted for age, sex, smoking habits and educational level. There was no association between chronic bronchitis or emphysema and indices of traffic air pollution. On the other hand, self-reported intense traffic was associated with asthma prevalence, but there was no evidence of association when other measures of air pollution were examined. Finally, self-reported traffic levels, distance from HTRs, PM emissions, and estimated NO_2_ were all associated with rhinitis.

**Table 4:** Association between environmental exposures and respiratory diseases

	Chronic bronchitis or emphysema (n = 397)		Asthma (n = 472)		Rhinitis (n = 1227)	
	%	OR (95% CI)	%	OR (95% CI)	%	OR (95% CI)
Self-reported traffic						
Absent	4.2	1.00	4.3	1.00	11.1	1.00
Low	3.7	0.88 (0.64 to 1.20)	4.7	1.11 (0.83 to 1.48)	12.8	1.14 (0.95 to 1.38)
Intermediate	4.3	1.04 (0.77 to 1.40)	5.0	1.18 (0.88 to 1.58)	13.3	1.18 (0.98 to 1.42)
High	4.9	1.19 (0.84 to 1.69)	6.3	1.46 (1.05 to 2.03)	14.9	1.30 (1.05 to 1.62)
p trend		0.211		0.025		0.019
Distance from high traffic roads						
>200 m	4.4	1.00	4.9	1.00	12.3	1.00
100–200 m	4.0	0.89 (0.66 to 1.20)	4.9	1.00 (0.77 to 1.29)	12.8	1.01 (0.85 to 1.19)
50–100 m	3.1	0.69 (0.45 to 1.05)	5.3	1.07 (0.76 to 1.52)	15.4	1.26 (1.03 to 1.54)
<50 m	4.1	0.94 (0.67 to 1.31)	4.9	1.01 (0.73 to 1.39)	14.6	1.18 (0.96 to 1.44)
p trend		0.278		0.851		0.030
Metres of HTR within 200 m from home						
None	4.4	1.00	4.9	1.00	12.3	1.00
Low (<416 m)	3.9	0.90 (0.63 to 1.26)	5.4	1.09 (0.81 to 1.46)	13.9	1.09 (0.90 to 1.32)
Medium (416–798 m)	3.5	0.75 (0.52 to 1.07)	5.7	1.16 (0.87 to 1.54)	14.3	1.16 (0.97 to 1.39)
High (>798 m)	4.1	0.94 (0.69 to 1.29)	4.0	0.80 (0.58 to 1.11)	13.6	1.09 (0.90 to 1.32)
p trend		0.285		0.572		0.152
Quartiles of PM emissions						
1st	4.2	1.00	4.9	1.00	10.6	1.00
2nd	4.1	0.96 (0.71 to 1.30)	5.4	1.10 (0.84 to 1.44)	14.7	1.41 (1.17 to 1.69)
3rd	4.1	0.90 (0.66 to 1.23)	4.7	0.94 (0.71 to 1.24)	12.1	1.11 (0.92 to 1.34)
4th	4.5	1.05 (0.77 to 1.42)	5.2	1.06 (0.80 to 1.39)	14.7	1.37 (1.14 to 1.64)
p trend		0.871		0.980		0.018
Estimated NO_2_ (quartiles, μg/m^3^)						
1st (21.0–37.3)	4.2	1.00	4.6	1.00	10.8	1.00
2nd (37.3–47.3)	4.6	1.03 (0.77 to 1.39)	5.0	1.11 (0.84 to 1.47)	13.3	1.27 (1.06 to 1.53)
3rd (47.3–50.3)	3.9	0.90 (0.65 to 1.23)	5.1	1.08 (0.81 to 1.44)	13.2	1.15 (0.96 to 1.39)
4th (50.3–62.6)	4.2	0.97 (0.71 to 1.31)	5.2	1.11 (0.84 to 1.48)	14.7	1.30 (1.08 to 1.56)
p trend	0.624	0.624		0.532		0.020

OR, odds ratio adjusted for age, sex, smoking habit and educational level.

Table 5 shows the association between the score indicator of traffic pollution exposure and respiratory diseases for the entire study population, and among non-smokers and smokers. There was no association between traffic exposure and chronic bronchitis or emphysema and asthma in the entire population as well as among non-smokers and smokers. However, with higher exposure scores, there were greater odds of suffering from rhinitis, with a statistically significant trend, both for the entire study population and for non-smokers. There was no association between traffic exposure and rhinitis in those who smoke (p value for the interaction term = 0.083). When we analysed the association between score indicators of traffic exposure and respiratory diseases stratified by educational level, there was no statistically significant interaction for any of the outcomes in the study, but the association between traffic-related air pollution and rhinitis was more evident among adults with low and medium educational levels than among those with high education (data not shown).

**Table 5:** Association between score indicator of traffic pollution exposure and respiratory diseases, by smoking habit

	n	Chronic bronchitis or emphysema		Asthma		Rhinitis	
		%	OR (95% CI)	%	OR (95% CI)	%	OR (95% CI)
All study population							
Very low	2175	4.2	1.00	4.6	1.00	10.9	1.00
Low	2131	4.5	1.05 (0.77 to 1.44)	5.4	1.23 (0.92 to 1.64)	12.6	1.14 (0.94 to 1.38)
Intermediate	2668	4.1	0.94 (0.69 to 1.27)	5.0	1.11 (0.83 to 1.48)	13.9	1.22 (1.01 to 1.46)
High	2322	4.1	0.93 (0.68 to 1.28)	4.9	1.07 (0.79 to 1.44)	14.3	1.28 (1.06 to 1.54)
p trend			0.513		0.887		0.009
Non-smokers							
Very low	1279	3.0	1.00	4.9	1.00	11.6	1.00
Low	1242	3.2	0.99 (0.63 to 1.57)	6.2	1.30 (0.91 to 1.87)	14.4	1.24 (0.97 to 1.58)
Intermediate	1621	3.4	1.06 (0.68 to 1.64)	4.9	1.01 (0.70 to 1.45)	16.6	1.43 (1.14 to 1.80)
High	1408	3.5	1.08 (0.69 to 1.69)	5.6	1.19 (0.83 to 1.72)	16.8	1.50 (1.19 to 1.89)
p trend			0.693		0.671		<0.001
Smokers							
Very low	896	5.8	1.00	4.0	1.00	9.8	1.00
Low	889	6.3	1.08 (0.71 to 1.65)	4.4	1.09 (0.68 to 1.76)	10.0	0.98 (0.71 to 1.34)
Intermediate	1047	5.3	0.87 (0.58 to 1.31)	5.3	1.28 (0.81 to 2.03)	9.7	0.88 (0.64 to 1.21)
High	914	5.0	0.82 (0.53 to 1.27)	3.7	0.85 (0.50 to 1.44)	10.4	0.92 (0.67 to 1.28)
p trend			0.240		0.726		0.522

OR, odds ratio adjusted for age, sex, smoking habit and educational level.

## DISCUSSION

This study shows that different indices of exposure to traffic air pollution in the city of Rome were moderately correlated to one another. The exposure indices were consistently associated with prevalence of rhinitis, while only self-reported traffic density was associated with asthma prevalence, and there were no exposure indices associated with chronic bronchitis. Combining the indices into an exposure score gave the best fit to the data and indicated that the association between rhinitis and traffic-related air pollution was limited to non-smokers.

Only a few studies have evaluated the correlation between different types of exposure to traffic-related air pollutants.^7^ ^21^ ^22^ Heinrich and colleagues analysed subjective measures of traffic intensity and GIS-modelled exposures in the Netherlands and in Munich. They found slightly higher NO_2_ and PM_2.5_ estimated levels with self-reports of high traffic at home in Munich and in urban Dutch areas, while no association between self-report and measured air pollution levels were found for rural Dutch areas. The authors did not find an association between socioeconomic position and estimated air pollution, while in Rome, due to historical urban development, higher levels of air pollution in high socioeconomic areas have been found in this and in a previous study.^22^ ^23^ 

The SAPALDIA study used different GIS measures to investigate the association between respiratory symptoms and traffic air pollution.^7^ The authors found an increased risk of regular phlegm for those living within 20 m of a main street, and an increased risk of attack of breathlessness per 500 m increase of length of main streets within 200 m from home. In never smokers, they found that attack of breathlessness and wheezing without a cold was related to the length of main streets within 200 m from home, and that wheezing with breathing problems was related to living within 20 m of a main street. In our study we did not find an association between length of HTR within 200 m from home and respiratory problems, while adults were more likely to suffer from rhinitis with decreasing distance from HTRs, increasing levels of PM and NO_2_ at the home address, as well as increasing self-reported traffic intensity.

The increased risk of asthma with higher self-reported traffic density in our study was not confirmed by more objective air pollution measures. The simplest explanation may be reporting bias, as suggested by Kuehni and colleagues,^14^ with a higher probability of reporting a high traffic exposure for asthmatic subjects. However, it is plausible that asthmatics are more sensitive than non-asthmatics to air pollution and to other of the respiratory system’s irritating factors, and have a different perception of the level of traffic than the general population. In addition, given the available evidence regarding the short-term effects of air pollution on asthmatics,^24^ it is also plausible that diseased subjects are directly affected from air pollution peaks, thus severity increases together with an increased perception of the exposure although the overall prevalence remains unchanged. A reason for the inconsistent results for asthma is the lack of specific information about time of onset of the disease and about the presence of current symptoms.

It is well accepted that air pollution from traffic, especially diesel emissions, might influence asthma and rhinitis enhancing immunological responses to allergens, and induce inflammatory reactions in the airways at relatively low concentrations and even with short exposure durations.^25^ Animal studies showed that exposure to diesel exhaust particles in mice enhances airway inflammation, hyper-responsiveness, and IgE antibody responses.^26^ ^27^ In vitro studies on human bronchial epithelial cells, the first line of cellular defence against inhaled irritants, suggested that diesel exhaust particles might modulate airway disease influencing them.^28^ Laboratory studies show that increased exposures to NO_2_, ozone, and PM may play a role in both allergic and non-allergic respiratory diseases.^29^

Although there is a good support from experimental evidence, the results of epidemiological studies are not so clear.^6^ Most of the studies on the effects of air pollution on respiratory health have been conducted on children, and a number of them suggest that air pollution is associated with allergic rhinitis.^4^,^5^,^30^^–^32 Findings among adults have been equivocal.^6^,^7^,^9^,^33^,^34^ A time series study reported that air pollution worsens allergic rhinitis symptoms, leading to substantial increases in doctor consultations.^33^ In the SAPALDIA study, an association between exposure to traffic and allergic sensitisation was detected.^7^ However, no association was found with symptoms of hay fever. Heinrich and colleagues found an association between chronic bronchitis and traffic-related air pollutants, but they did not find any association with hay fever and wheezing in the non-smoker population.^9^ Our results on rhinitis are then of particular interest. We found that current smoking is inversely associated with rhinitis, a result that has been observed before and explained on the basis of a “healthy smoker effect” or decreased susceptibility to the effect of seasonal allergens.^35^ However, the effect of traffic air pollution on rhinitis in our study was found in non-smoking adults, a result that may suggest that the allergic mechanism may be specifically vulnerable in non-smokers or that the response to irritation from air toxicants may be enhanced.

**Main messages**

- The correlation between self-reported traffic intensity and more objective categorical indices of exposure range from 0.32 to 0.49.
- All indices of exposure to traffic air pollution were directly associated with socioeconomic position.
- Rhinitis was strongly associated with traffic-related air pollution.

**Policy implication**

Rhinitis is related to traffic air pollution in adults; efforts should be made to reduce exposure.

The main strengths of the study are the large size and the rich set of exposure indicators that we have analysed, yet there are some weaknesses to note. The main weakness is that chronic bronchitis, asthma and rhinitis were self-reported. In this, as in other studies, no objective measurements were taken.^3^^–^5 Moreover, the way the questions were formulated did not allow us to understand the timing of disease onset, or if they were recent or past problems. This is likely to bias the results toward the null, and could be the reason we did not find any association between traffic-related air pollution and asthma. In addition, the measures of air pollution that we used are estimated at the residence of participants; they do not take into account the amount of time the subjects spent at home or in the area of residence, and they are not indoor measures. Furthermore, data on PM emissions were collected during 2002, while the cross-sectional study was carried out in 1995. To study the effects of long-term exposure to traffic air pollution we selected subjects that had lived in the same place for at least 3 years at the time of the interview, but we could not assess the possibility that subjects may have moved before, possibly because of their disease.

In conclusion, the evaluation of several exposure indicators suggests that rhinitis in adults is linked to exposure to traffic air pollution. The strongest effect was found using NO_2_ from a land-use regression model but the combination of the various exposure indicators in a summary score gave the best dose–response slope.
